# Electrocardiographic Patterns of Depolarization Abnormalities Help to Identify Reduced Left Ventricular Ejection Fraction

**DOI:** 10.3390/diagnostics12082020

**Published:** 2022-08-21

**Authors:** Maria Gordeeva, Irina Serdiukova, Alexander Krasichkov, Elena Parmon

**Affiliations:** 1 Federal State Budgetary Institution “Almazov National Medical Research Centre” of the Ministry of Health of the Russian Federation, 197341 Saint Petersburg, Russia; 2 Radio Engineering Systems Department, Saint Petersburg Electrotechnical University “LETI”, 197022 Saint Petersburg, Russia

**Keywords:** fragmentation, early repolarization pattern, chronic heart failure, ECG, CHF, mildly reduced ejection

## Abstract

The aim of the study was to investigate the relationship between a decrease in the left ventricular ejection fraction (EF) and traditional ECG signs associated with structural changes of the myocardium (pathological Q wave, ventricular arrhythmias) and relatively new and poorly understood (fragmented QRS complex (fQRS), early repolarization pattern (ERP)) and evaluate their significance for identifying patients with mildly reduced EF (mrEF). The study included 148 patients who were treated and examined at the Almazov Medical Research Center. FQRS, ERP, pathological Q wave, and premature ventricular contractions (PVC) were described in the analysis of the ECG, and the results of echocardiography and statistical data were analyzed: Fisher’s test and chi-square, correlation analysis, and ROC analysis. According to the level of EF, patients were divided into three groups: group 1—patients with low EF (lEF) (less than 40%), group 2—patients with mildly reduced EF (mrEF) (40–49%); group 3—patients with preserved EF (pEF) (more than 50%). In the first group (EF), fQRS was registered in 16 (51.6%) patients, in the mrEF in 16 (18.2%). Pathological Q wave was detected in lEF in 20 (65%), in mrEF in 10 (35%), 15 (18%), in pEF in 15 (18%). The fQRS has been found to be more important in identifying patients with mrEF. In lEF in 2 (6.5%) patients, in mrEF in 2 (6.9%), in pEF in 11 (12.5%). There was no relationship between ERP, the amount of PVC, and the presence of ventricular tachycardia with EF. FQRS is significantly more common occurred with a decrease in EF and may be a marker of a mrEF. Thus, fQRS is associated with mrEF and pay close attention in routine clinical practice to identify patients at high risk of developing systolic dysfunction.

## 1. Introduction

In 1997, the prominent cardiologist Eugene Braunvald called chronic heart failure (CHF) a new epidemic [1]. Over the past 20 years, in spite of considerable achievements in CHF treatment, the number of such patients in the population has remained rather high (8.2% in Russia, according to the EPOCH-CHF study [2]) and continued to increase. Such a widespread CHF is associated with an increase in the age of the population as a whole and an increase in risk factors such as LV hypertrophy, diabetes, and hypertension. The number of patients with coronary artery disease and cardiomyopathies is growing, including due to toxic effects (smoking, alcoholism, drug addiction), as well as due to drug therapy, for example, the almost massive use of antipsychotics, the use of which is associated with ventricular arrhythmias and CHF [3]. CHF also remains one of the frequent causes of hospitalization and makes a significant contribution to mortality [4]. Identifying patients with CHF, especially at the early stages, is an extremely important yet unresolved task [5,6].

One of the primary criteria for CHF diagnosis is left ventricular ejection fraction (EF), which predetermines further treatment tactics. For a long time, EF under 40% (lEF) had been considered significantly low, but in 2016 the European Society of Cardiology suggested distinguishing a group of patients with mildly reduced EF (mEF)—40–49% [7]. Now it is this group that has attracted much attention, as research has shown the relevance of early identification and start of treatment of CHF with mEF [8,9,10,11,12]. However, clinical manifestations of CHF in such patients are much less evident, which complicates timely diagnosis and treatment.

The most popular method for CHF diagnosis and EF evaluation is echocardiography, but it is not primary for screening examination of patients either in the general population or among patients with known heart diseases. Electrocardiography (ECG) remains the most widely used method for cardiovascular examination in all groups of patients. However, ECG signs that are observed in the case of reduced EF (increased duration of QRS complex, increased PR, QT intervals, His bundle block), and other patterns of structural changes in the myocardium are potentially associated with reduced EF (pathological Q wave, left ventricular hypertrophy signs, poor R wave progression, etc.) are non-specific [13,14,15,16,17].

There are some studies that have investigated the diagnostic accuracy of ECG as a method allowing to suggest reduced EF. Thus, the meta-analysis carried out by K. Khunti et al. (2004) demonstrated a low diagnostic accuracy of ECG in identifying reduced EF (sensitivity from 73% to 94%, and specificity from 20% to 65%). The present study accounted for such traditional non-specific signs as His bundle block, duration of the QRS complex, signs of previous myocardial infarction, poor R wave progression, LV hypertrophy signs [18]. It should be noted that most investigations into ECG as a method for screening identification of reduced EF focused on EF below 40%. Identifying mEF by ECG appears to be a more complicated task. Researchers are now looking for new ECG patterns and approaches (such as index development and use of artificial intelligence) for the diagnosis of heart failure, including that with mildly reduced or preserved ejection fraction, investigating such indices as PR and QT interval dispersion, Tpeak/Tend relation, QRS-T-angle, etc. [19].

The most important factor in the occurrence and progression of LV systolic dysfunction is cardiac fibrosis. It is known that both focal and interstitial fibrosis can primarily lead to disorders of myocardial depolarization [20]. Therefore, ECG signs that reflect depolarization disorders, particularly fragmented QRS complexes (fQRS) and early repolarization pattern (ERP), as well as premature ventricular contractions (PVC), are the most promising ECG signs for detection of reduced EF [21].

The association of fQRS with cardiac fibrosis has been proven in a number of studies [22,23,24]. The risk stratification significance of this ECG sign has been demonstrated for patients with various cardiovascular pathologies: ischemic heart disease [25,26,27,28,29], hypertrophic cardiomyopathy [30,31], Brugada syndrome [32], and CHF with reduced EF [33]. The relationship between fQRS and myocardial systolic dysfunction has been studied to a lesser extent. It is known that in patients with chronic kidney disease, fQRS in inferior leads is associated with reduced EF [34]. As shown by Nikoo et al. (2020) and Bayramoğlu et al. (2019), fQRS can be a predictor of reduced EF in healthy individuals [35,36]. As regards the ERP, its association with structural changes in the myocardium has been demonstrated, and a correlation with posterior wall thickness has been found [37,38,39]. High-risk stratification significance of ERP was found in patients with myocardial structural changes of various genesis [40] and in the general population [41], as well as in CHF patients [42]. However, the correlation of this ECG sign with a reduced ejection fraction of the left ventricular has not been studied sufficiently.

Therefore, it seems worthwhile to study fQRS and ERP in the context of reduced EF primary screening.

The purpose of our study was to investigate the correlation of reduced EF with traditional ECG patterns associated with structural changes in the myocardium (pathological Q wave, QRS complex duration, ventricular arrhythmias) and relatively new and less studied ECG patterns of depolarization disorders (fQRS, ERP), and to estimate their predictive value for identifying patients with mEF.

## 2. Materials and Methods

The study included 148 patients who underwent examination and treatment at the Almazov Medical Research Center, with various myocardial structural changes in ischemic and non-ischemic genesis. Retrospectively, we analyzed anamnestic data and data of cardiac visualization methods (echocardiography, magnetic resonance tomography, single photon emission computed tomography (SPECT)), ECG, and 24 h Holter monitor.

A total of 75.6% of the examined structural changes in the myocardium were associated with ischemic disease. The majority of patients (87.1%) had a diagnosis of hypertension. Less than half of the patients (37.1%) had a typical clinical presentation of angina pectoris, with the most frequently observed symptoms at level II class. Manifestations of CHF were detected in 66.4% and were more often noted in II class (NYHA). The studied groups, when it was divided by the level of EF, were comparable in clinical characteristics. According to the literature data, there was no relationship between the presence of QRS fragmentation and ERP drug therapy, differences in the therapy taken in the study groups were not considered.

The study was carried out in the framework of the large research project “Development of new technologies for prevention and neuromodulation-based treatment of heart failure”, under agreement no. 075-15-2020-800 of 24.09.2020 between the Almazov Medical Research Center and the Ministry of Science and Higher Education of the Russian Federation.

In all patients, we analyzed the results of 24 h Holter ECG monitoring and ECG in 12 leads at standard settings (12-channel ECG recording: high-frequency filter: 0.05–20 Hz, low-frequency filter: 100–150 Hz, paper speed: 25–50 mm/s, voltage: 10 mm/mV). Electrocardiograms were analyzed by two researchers independently. For fQRS, we used criteria proposed by Das et al., according to which fragmentation meant two or more notches on R or S waves at least in two contiguous leads in narrow complexes and three or more notches, or only two notches—with the distance between them over 40 ms [43]. Criteria for ERP were the presence of a QRS complex slur or notch on the descending knee of the R wave above the baseline. The J peak should be more or equal to 0.1 mV in two or more contiguous leads, except for leads V1–V3. The ERP was evaluated only in narrow complexes (below 120 ms) [44]. Localization of identified changes was evaluated for EVR and fQRS: changes registered in leads V1–V3 corresponded to the LV anterior wall, changes in leads I, aVL, and V6 corresponded to the lateral wall, changes in leads II, III, and aVF corresponded to the inferior wall. The maximum duration of the QRS complex and the presence of a pathological Q wave were also evaluated by ECG. The number of PVC s and the presence of ventricular tachycardia (VT) were evaluated by 24 h Holter monitor.

For all patients, we analyzed the results of echocardiogram performed on VIVID 7 Dimension devices (General Electric, Boston, MA, USA) using the standard protocol in accordance with recommendations of the European Society of Echocardiography. We analyzed parameters reflecting the left ventricular systolic function, such as end-diastolic size (EDS), end-systolic size (ESS), EF (according to Simpson), end-systolic volume (ESV), end-diastolic volume (EDV), and interventricular septum thickness (IVS).

## 3. Statistical Assessment

Initially, we conducted an exploratory analysis of categorical and quantitative variables of the database to group patients by EF, built a mathematical model, and calculated characteristics of variational series: expected value, dispersion, standard deviation, variation coefficients, skewness, excess, and other statistical data, and also conducted gap analysis. The analysis of quantitative variables was carried out using a one-way analysis of variance. Then, for quantitative variables, we analyzed distribution by the Kolmogorov–Smirnov one-sample test. The Kolmogorov–Smirnov test was used to check distributions for normality. For the selected quantitative variables, interrelation with the target variable was determined by using nonparametric methods according to the Mann–Whitney U test. Following this analysis, we selected quantitative variables that could be potential predictors. For nominal variables, interrelation with the target variable was verified with the chi-square and Fisher tests. Based on these tests, potential predictors were determined from nominal variables. The next step in statistical analysis was to build a logistic regression model.

We built and analyzed four models: stepwise exclusion of predictors and stepwise inclusion of predictors, each considered with and without permanent inclusion. The most significant was the model built by using the stepwise inclusion method. This model variant was selected for building by the forced inclusion method. The quality of the built model was determined by using Nagelkerke R2 coefficients.

In this study, we also used ROC curves to analyze the impact of the pathological Q wave and leads in which fQRS was registered in the group with mEF.

## 4. Results

Patients were divided into three groups according to the ejection fraction level based on echocardiography data: Group 1—patients with low EF (lEF) (below 40%): total 31 (25 males, average age 52.0 +/− 15.6); Group 2—patients with mildly reduced EF (mEF) (49–40%): total 29 (23 males, average age 54.7 +/− 12.4); Group 3—patients with preserved EF (pEF) (over 50%): total 88 (57 males, average age 58.2 +/− 12.0)—control group.

**Figure 1:**
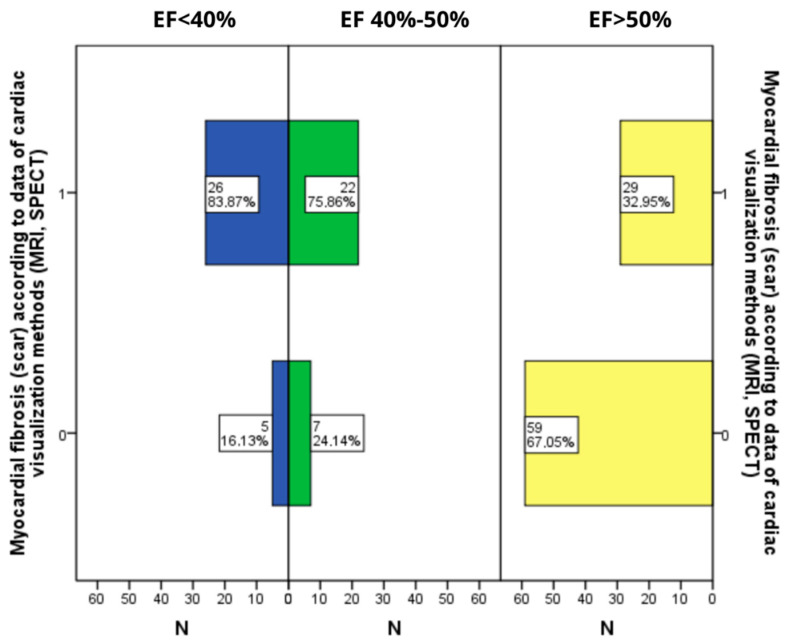
N—number of observations, EF—ejection fraction of the left ventricular, MRI—magnetic resonance imaging, SPECT—single photon emission computed tomography; 0—there is no fibrosis (scar); 1—there is fibrosis (scar).

In the group of patients with pEF, fibrotic changes (post-infarction and post-myocarditis fibrosis) were significantly rarer (*p* < 0.001), while there were no statistically significant differences in the occurrence of such structural changes in patients with mEF and lEF (Figure 1).

In group 1 (lEF), fQRS was registered in 16 patients (51.6%); in group 2 (mEF)—in 13 patients (44.8%), in group 3 (EF over 50%)—in 16 patients (18.2%).

**Figure 2:**
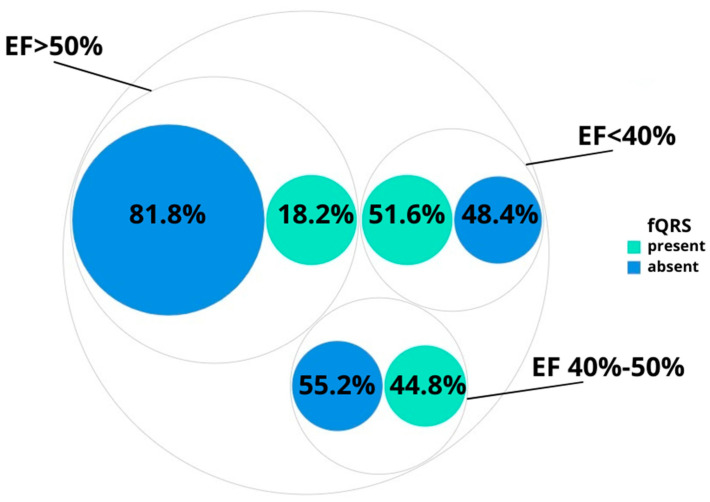
The occurrence of fQRS in the groups under study; EF—ejection fraction, fQRS—fragmented QRS complex.

Figure 2 shows the distribution of the observation number for the fQRS parameter in the groups under study. Indices were compared by χ2 tests, and differences were statistically significant (*p* < 0.001).

**Figure 3:**
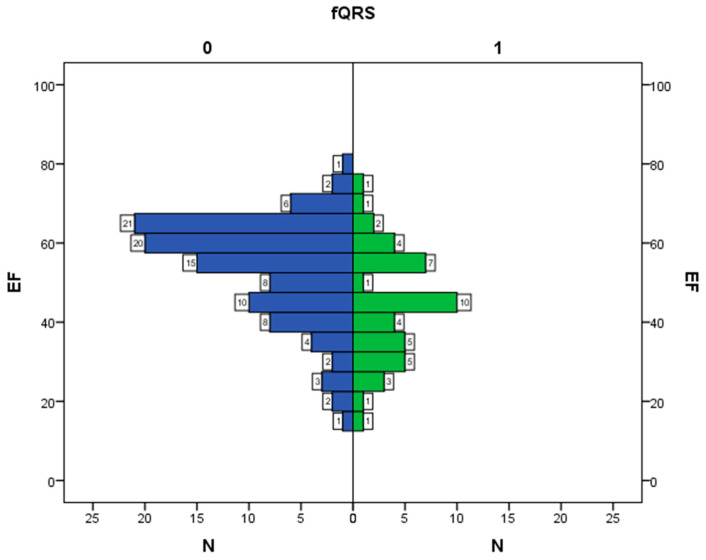
Interrelation between ejection fraction and fQRS; N—number of observations, EF—ejection fraction, fQRS—fragmented QRS complex, 0—there is no fragmented QRS complex, 1—there is fragmented QRS complex.

Figure 3 shows the histograms of EF distribution relative to values of the fQRS variable, which allows evaluating in detail the number of observations for each EF value. The result is statistically significant (*p* < 0.001).

**Figure 4:**
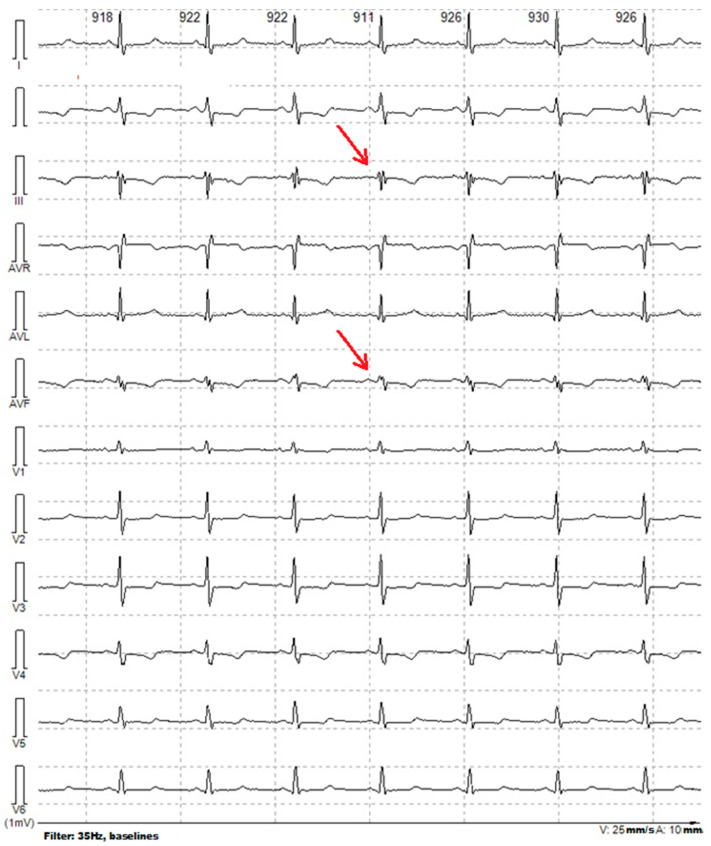
Example of ECG with fQRS in a female patient with mildly reduced ejection fraction of the left ventricular; FQRS in leads III and AVF, no pathological Q wave was registered, the QRS duration is normal; Arrows shows the fragmentation of the QRS.

Figure 4 shows an example of ECG with fQRS in leads III and AVF in the female patient G. (37 years old) with mEF (43%), without myocardial fibrosis according to echocardiographic and cardiac MRI data.

ECG leads were analyzed in all the groups in which fQRS was registered. The first group of patients was characterized by the presence of fQRS in ECG leads of the LV anterior wall, the second group of patients—the LV lateral wall, and the third group—the LV inferior wall. The results are presented in Table 1.

We analyzed the correlation between fQRS and such indices of the myocardial systolic function as EF, EDV, ESV, EDS, ESS, and IVS thickness. The results are presented in Table 2.

**Figure 5:**
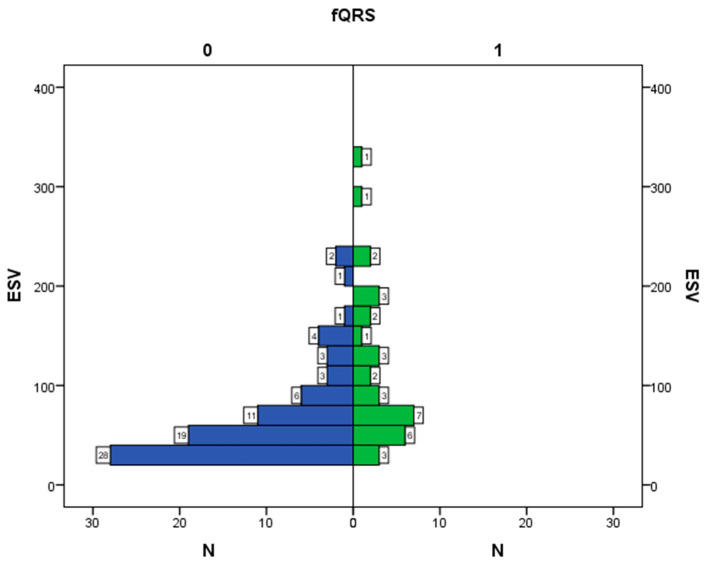
Interrelation between fQRS and end-systolic volume; N—number of observations, fQRS—fragmented QRS complex, ESV—end-systolic volume, 0—there is no fragmented QRS complex, 1—there is fragmented QRS complex.

Weak direct correlation between fQRS and EDV, ESV, ESS, VRD, and EF. Figure 5 shows the interrelation between fQRS and ESV. In patients without detected fQRS, ESV was observed within the range of 20–80 mL. In patients with fQRS, ESV was within the range of 40–80 mL. Values were compared by the Mann–Whitney U test, and the differences were statistically significant (*p* < 0.001).

**Figure 6:**
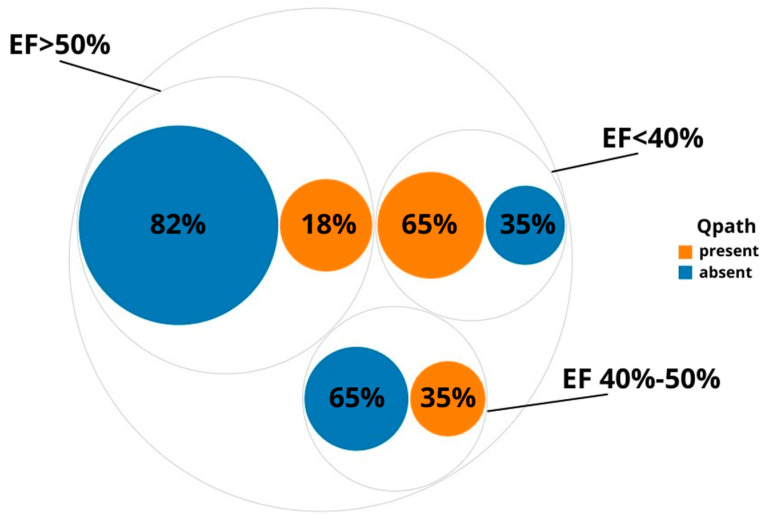
The occurrence of pathological Q wave in the groups under study; Qpath—pathological Q wave.

Pathological Q waves were found in 20 patients (65%) of group 1 (lEF); in 10 patients (35%) of group 2 (mEF); in 15 patients (18%) of group 3 (EF over 50%). Values were compared by the χ2 tests, and differences were statistically significant (*p* < 0.001) (Figure 6).

In patients with mEF, interrelation was found between the presence of fQRS and leads in which it was registered (marked gray); no interrelation was found with other parameters (Table 3).

Based on the data obtained, a logistic regression model was built using the forced inclusion method. For the resulting logistic regression model, a pair of expressions describes the probability of the predicted mFV p:

p = 1/(1 + exp(−z)),(1)

z1 = 1.849 − 2.185 × x1 + 4.940 × x2 + e,(2)

where z1 is the regression Equation (2) for the observed sample, x1 is the fQRS variable; x2—variable the occurrence of fibrosis changes according to data of cardiac visualization methods (MRI, SPECT); e—random errors of model building.

**Figure 7:**
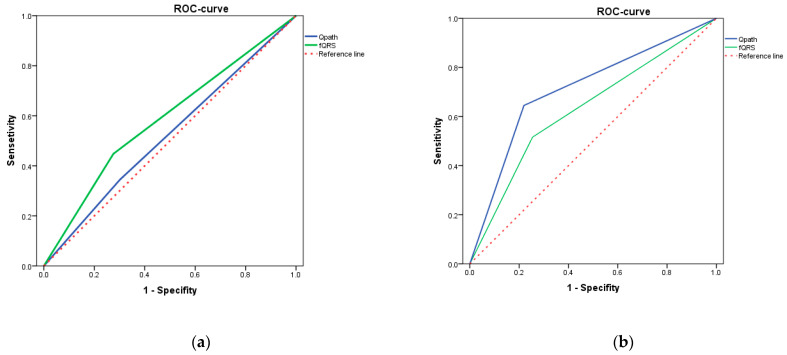
ROC curves for pathological Q wave and fQRS (**a**) in patients with mildly reduced EF; (**b**) in patients with lEF. Qpath—pathological Q wave, FQRS—fragmented QRS complex.

The assessment of ROC curves showed that fQRS was of greater importance for identifying patients with mEF than Q wave in patients with mEF (Figure 7a). Based on the ROC analysis results in patients with lEF, the Q wave had the highest predictive value, not fQRS (Figure 7b). The screening value of each marker for Figure 7 can be found in Table 4.

The occurrence of the ERP in the groups under study was analyzed. In group 1 (lEF), ERP was registered in 2 patients (6.5%); in group 2 (mEF) in 2 patients (6.9%); in group 3 (EF over 50%) in 11 patients (12.5%). Values were compared by the χ2 tests, and differences were not statistically significant (*p* = 0.5).

The occurrence of ERP was studied depending on EF; no correlation between EVR and EF was found by Spearman rank correlation analysis (correlation coefficient = 0.155).

ECG leads were analyzed in all the groups in which ERP was registered. The results are presented in Table 5.

We also analyzed the differences in the ERP morphology (slur or notch) depending on the group under study. In group 1, the ERP pattern with a notch was found in one patient (50%), and the EVR pattern with a wave was found in one patient (50%). In group 2, a notch was found in one patient (50%), and a wave was found in one patient (50%). In group 3, a notch was found in 2 patients (18.2%), and a wave was found in 9 patients (81.8%). Differences outside the groups were not statistically significant.

We analyzed the relationship between such indices of EF, EDV, ESV, EDS, ESS, IVS thickness, and ERP. No significant correlations were found.

**Figure 8:**
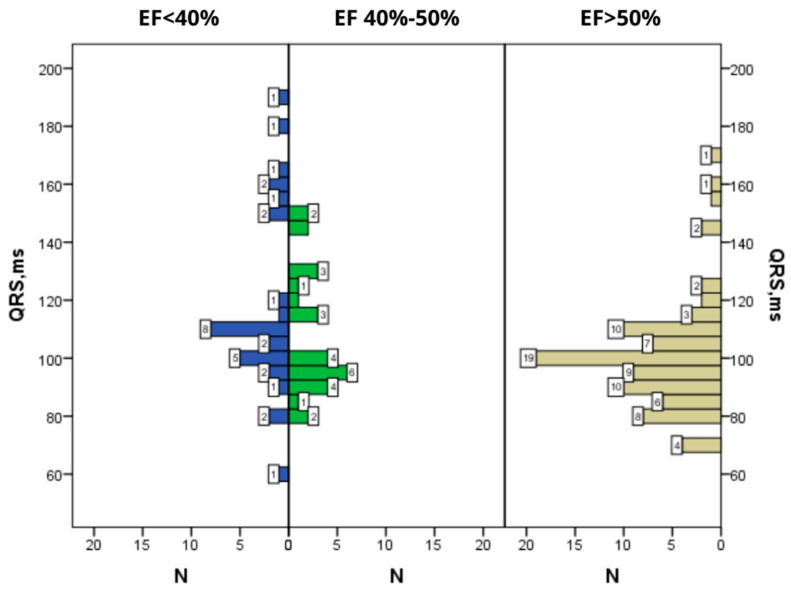
Differences between the groups by the duration of QRS complex; N—number of observations, EF—ejection fraction, fQRS—fragmented QRS complex.

The distribution of patients in the studied groups was analyzed depending on the duration of the QRS complex (Figure 8). The QRS duration is inversely dependent on EF, which follows from the mean value (ms) and confidence interval. The results are presented in Table 6.

The average number of PVC in patients under study was 2685.16 per day, the root-mean-square deviation was 7119, which means that there was a wide range of VEC values among patients—from 0 to 36,721. No correlation was found between the number of VECs and EF (correlation coefficient = −0.058). The result is statistically significant (*p* = 0.16).

Correlation analysis did not reveal any correlation between EF and VT (correlation coefficient = −0.251). ROC curve analysis did not show trustworthy results either.

The obtained data for the three groups are visually represented in summary Table 7.

Confidence interval 95% (top and bottom values) (ms)

In general, this study analyzed the relationship between ECG signs that reflect depolarization disorders and parameters that characterize left ventricular dysfunction. The statistical analysis of fQRS in the groups under study showed that this ECG sign is generally absent in patients with preserved EF. It was found that in 62.5% (10) patients, fQRS was registered in the leads corresponding to the LV inferior wall. The statistical analysis of ERP in the groups under study showed similar values for groups with low and mildly EF, and in most patients, the early ventricular repolarization pattern was absent. A pathological Q wave was more often registered in patients with low EF (group 1). A logistic regression model was used to check whether it would be possible to predict the potential inclusion of patients into the group with mildly EF. As shown by performed analysis, fQRS had the highest predictive value for identifying patients with IEF.

Thus, fQRS was significantly more often observed in the case of reduced ejection fraction—both in the group with mEF and in the group with lEF. The same dependence was peculiar to both a pathological Q wave and increased duration of the QRS complex. However, in general, it was FQRS that proved to be a more representative marker than a pathological Q wave with regard to identifying reduced EF. The ERP pattern was much rarer in patients in the groups under study; no statistically significant correlation was found between EVR and EF, nor was any correlation found between VRD and LV systolic dysfunction.

## 5. Discussion

This study analyzed the relationship of reduced EF with both the traditional ECG signs associated with structural changes in the myocardium (pathological Q wave, QRS complex duration, ventricular arrhythmias) and relatively new and less studied ones (fQRS, ERP).

Thus, in our study, a pathological Q wave was more often registered in patients with lEF (almost twice more often than in patients with mEF), which is consistent with some other studies that also demonstrated the association of this ECG sign with reduced EF below 40% [45,46]. The presence of a pathological Q wave in ECG often points to existing myocardial fibrosis. Increased duration of QRS complex is also associated with structural changes in the myocardium: according to literature, QRS of over 120 ms in patients with reduced EF has been found in 14–47% of cases [47]. In our study, the largest duration of the QRS complex was found in patients with EF below 40% and exceeded 110 ms. Both in patients with mEF and in patients with pEF, the duration of the QRS complex was predominantly within the range of 80 to 100 ms. Thus, neither the presence of a pathological Q wave nor the duration of the QRS complex allowed us to identify a group of patients with mEF.

Although ventricular arrhythmias (PVC, VT) are not ECG signs pointing to structural changes in the myocardium and reduced EF, they are often considered in connection with CHF as a manifestation of changed depolarization associated with cardiac fibrosis [48]. However, we did not find any correlation between the quantity of PVCs, the presence of VT, and reduced EF. Reduced EF below 40% is commonly considered a high-risk factor for sudden cardiac death (SCD), but only 13% of SCD cases occur in patients with lEF [49]. Numerous recent studies [50,51,52,53,54] argue that EF only is not sufficient for risk stratification of ventricular arrhythmia development, and our findings confirm this view.

Our study has established a relationship between the presence of fQRS as a relatively new ECG sign and left ventricular myocardial systolic dysfunction. Most researchers are currently considering fQRS as a marker of a high risk of arrhythmic events and SCD, including in patients with lEF, and it is also an ECG sign associated with cardiac fibrosis of different genesis. We have found only a few studies in which fQRS was considered a sign of reduced EF. For instance, Zhao et al. obtained results similar to our findings: in patients with myocardial infarction, the presence of fQRS was associated with an increased level of brain natriuretic peptide and reduced EF [55]. A recent study (2020) demonstrated the association of fQRS with regional left ventricular diastolic dysfunction [35].

Even though the occurrence of the ERP pattern in ECG can be caused by structural changes in the myocardium, we did not find any relationship between the presence of this ECG pattern and reduced EF. The ERP pattern should probably be viewed first of all as a marker of the electric instability of the myocardium, pointing to a high risk of ventricular rhythm disorders but not to systolic dysfunction of the left ventricular.

An important part of our study was devoted to finding ECG signs that could point to mildly reduced EF. In spite of the fact that a pathological Q wave was most often registered in the groups under study, it was fQRS that turned out to be a marker to a large extent associated with mEF.

## 6. Conclusions

ECG remains a leading screening method that allows suspecting a cardiovascular pathology. Yet traditional ECG signs are not sufficient for identifying patients with a mildly reduced ejection fraction of the left ventricular. Attention should be paid to patients with revealed fQRS, which could be not only a marker of a high risk of ventricular rhythm disorders or structural changes in the myocardium but also a sign pointing to a moderately reduced EF.

General practitioners should thoroughly study the ECG in the clinical routine, educating clinicians to pay attention to the presence of QRS fragmentation and to suspect that such patients may have early-stage CHF. The evaluation of these ECG markers can be performed manually and does not require any special skills. Moreover, our study, once again proving the importance of determining fragmentation, once again calls for a wider introduction of this marker into routine clinical practice.

Among asymptomatic and oligosymptomatic patients, the most common method of examination is precisely ECG. Thus, after identifying the pattern of fragmentation on the ECG, we can decide on further methods of examination-performing ECHO for the presence of myocardial dysfunction indicating rEF or determining BNP, which is not a screening method for assessing the state of the cardiovascular system in the general population.

Risk stratification of patients with lEF is also an extremely important issue now. Our findings confirm the need for further investigation into the risk stratification significance of known markers of electrical instability of the myocardium and the development of new approaches to risk stratification of patients with reduced EF, which are based not only on the level of reduced EF and the presence of ventricular arrhythmia but also on other significant predictive ECG signs.

## Figures and Tables

**Table 1:** Distribution of patients in the studied groups depending on localization of changes by leads in which fQRS was registered.

Localization of Changes	Group 1, n (%)	Group 2, n (%)	Group 3, n (%)
LV anterior wall	7 (43.75)	4 (30.70)	5 (31.25)
LV lateral wall	3 (18.75)	5 (38.46)	1 (6.25)
LV inferior wall	6 (37.5)	4 (30.70)	10 (62.50)

LV—left ventricular; Indices were compared by the χ2 tests, differences were not statistically significant (*p* = 0.5).

**Table 2:** Correlations between indices of the myocardial systolic function and fQRS.

		EF, %	IVS, mm	EDV, mL	ESV, mL	EDS, mm	ESS, mm
fQRS	Pearson correlation	−0.316	0.021	0.385	0.340	0.325	0.355
	Significance	<0.001	0.83	<0.001	<0.001	0.001	0.001
	N	148	108	119	112	110	85

EDS—end-diastolic size; ESS—end-systolic size; EF—ejection fraction; ESV—end-systolic volume; EDV—end-diastolic volume; IVS—interventricular septum thickness. Weak correlation: the correlation coefficient below 0.5; strong—above 0.75. N—number of observations

**Table 3:** Interrelation between studied ECG signs and mildly reduced ejection fraction of the left ventricular (40–49%).

	Pearson’s Chi-Squared Test	Fisher Test
fQRS	0.061	0.05
Leads with fQRS	0.033	
EVR	0.519	0.403
EVR morphology	0.642	
Leads with EVR	0.748	
Pathological Q wave	0.654	0.405
The occurrence of fibrosis changes according to data of cardiac visualization methods (MRI, SPECT)	<0.001	<0.001

FQRS—fragmented QRS complex, EVR—early ventricular repolarization; Marked gray—interrelation was found.

**Table 4:** Value of single-marker detection for patients with mEF and lEF.

	SEN	SPE	+LR	−LR	AUC
**Mildly reduced EF** **(Figure 7a)**					
Qpath	0.345	0.712	2.945	0.339	0.522
FQRSS	0.448	0.734	2.031	0.492	0.586
**Low reduced EF** **(Figure 7b)**					
Qpath	0.645	0.781	1.142	0.875	0.713
FQRSS	0.516	0.746	1.623	0.616	0.631

SEN—sensitivity; SPE—specificity; +LR: —positive likelihood ratio; −LR—negative likelihood ratio.

**Table 5:** Distribution of patients in the groups under study depending on ECG leads of ERP registration.

Localization of Changes	Group 1, n (%)	Group 2, n (%)	Group 3, n (%)
LV anterior wall	0	1 (50)	2 (20)
LV lateral wall	0	0	1 (10)
LV inferior wall	2 (100)	1 (50)	7 (70)

LV—left ventricular; values were compared by the χ2 tests, and differences were not statistically significant (*p* = 0.7).

**Table 6:** Distribution of patients in the studied groups depending on the duration of QRS complex.

	Group 1		Group 2		Group 3	
Average value (ms)	117.3		108.3		100	
MSD (ms)	30.9		21.8		18.6	
Minimal value (ms)	60		80		68	
Maximal value (ms)	190		152		172	
Confidence interval 95% (top and bottom value) (ms)	106	129	100	116.6	96	104
Number of observations	31		29		85	

MSD—mean square deviation; values were compared by the Mann–Whitney U test, and differences were statistically significant (*p* = 0.004).

**Table 7:** Summary for three EF groups.

			Group 1		Group 2		Group 3	
N			31		29		88	
fQRS	Registered		16 (51.6%)		13 (44.8%)		16 (18.2%)	
	Localization of changes	LV anterior wall	7 (43.75)		4 (30.70)		5 (31.25)	
		LV lateral wall	3 (18.75)		5 (38.46)		1 (6.25)	
		LV inferior wall	6 (37.5)		4 (30.70)		10 (62.50)	
Pathological Q wave (registered)			20 (65%)		10 (35%)		15 (18%)	
ERP	Registered		2 (6.5%)		2 (6.9%)		11 (12.5%)	
	Localization of changes	LV anterior wall	0		1 (50)		2 (20)	
		LV lateral wall	0		0		1 (10)	
		LV inferior wall	2 (100)		1 (50)		7 (70)	
	Morphology		wave	notch	wave	notch	wave	notch
			1 (50%)	1 (50%)	1 (50%)	1 (50%)	9 (81.8%)	2 (18.2%)
QRS	Average value (ms)		117.3		108.3		100	
	MSD (ms)		30.9		21.8		18.6	
	Minimal value (ms)		60		80		68	
	Maximal value (ms)		190		152		172	
	Confidence interval 95% (top and bottom values) (ms)		106	129	100	116.6	96	104
